# Determination of the electron-capture coefficients and the concentration of free electrons in GaN from time-resolved photoluminescence

**DOI:** 10.1038/srep37511

**Published:** 2016-11-30

**Authors:** M. A. Reshchikov, J. D. McNamara, M. Toporkov, V. Avrutin, H. Morkoç, A. Usikov, H. Helava, Yu. Makarov

**Affiliations:** 1 Department of Physics, Virginia Commonwealth University, Richmond, VA 23284, USA; 2 Department of Electrical and Computer Engineering, Virginia Commonwealth University, Richmond, VA 23284, USA; 3 Nitride Crystals, Inc. 181E Industry Ct., Ste. B, Deer Park, NY 11729, USA; 4 Saint-Petersburg National Research University of Information Technologies, Mechanics and Optics, 49 Kronverkskiy Ave., 197101 Saint Petersburg, Russia

## Abstract

Point defects in high-purity GaN layers grown by hydride vapor phase epitaxy are studied by steady-state and time-resolved photoluminescence (PL). The electron-capture coefficients for defects responsible for the dominant defect-related PL bands in this material are found. The capture coefficients for all the defects, except for the green luminescence (GL1) band, are independent of temperature. The electron-capture coefficient for the GL1 band significantly changes with temperature because the GL1 band is caused by an internal transition in the related defect, involving an excited state acting as a giant trap for electrons. By using the determined electron-capture coefficients, the concentration of free electrons can be found at different temperatures by a contactless method. A new classification system is suggested for defect-related PL bands in undoped GaN.

GaN is widely used in light-emitting devices such as blue and white LEDs and lasers^1^. It is also a very promising material for future high-power electronic devices^2^,^3^,^4^. In order to achieve high breakdown voltage, the concentration of point defects unintentionally introduced during the material growth must be significantly reduced. Unlike extended defects, which are easy to detect and identify, point defects in GaN are not well understood, and their detrimental role in electronic devices needs to be determined.

Hydride vapor phase epitaxy (HVPE) is a superior technique to obtain high-quality GaN with a low density of structural defects and a low concentration of point defects^5^. According to secondary ion mass-spectrometry (SIMS) analyses, undoped GaN grown by HVPE contains low concentrations of impurities. In particular, the concentration of carbon, which creates deep-level defects, can be reduced down to 10^15^ cm^−3^ (ref. 6), and the concentrations of oxygen and silicon (both acting as shallow donors in GaN) can be reduced down to 2 × 10^16^ and 2 × 10^15^ cm^−3^, respectively^7^,^8^,^9^. All these values are at the detection limit of SIMS measurements. To detect and quantify impurities and native defects with lower concentrations, photoluminescence (PL) can be used^10^. For this, the defect-related PL bands must be reliably identified and their characteristic parameters be found. Another challenge for the evaluation of the electrical properties of GaN grown by HVPE on sapphire substrates is the presence of a parasitic conductive layer near the GaN/sapphire interface^11^. This degenerate GaN layer shunts the conductivity of the bulk GaN and may introduce significant errors in the concentration of free electrons when it is determined with the Hall effect method at room temperature. The actual concentration of free electrons and their mobility in bulk GaN can be calculated by applying a two-layer model when the Hall effect is studied for a wide range of temperatures^12^. The concentration of uncompensated shallow donors in the vicinity of the GaN surface can also be estimated from capacitance-voltage measurements^13^. PL has an advantage over the electrical measurements as being a nondestructive method without the need for contact fabrication.

Recently, we have demonstrated that time-resolved PL can be a powerful tool to determine the concentration of free electrons and the concentration of point defects in semiconductors^14^. In order to find the concentration of free electrons from time-resolved PL measurements, the electron-capture coefficients (*C*_*nA*_) for the defect-related PL bands must first be found. The *C*_*nA*_ coefficient is an inherent property of a particular defect. Sometimes, broad PL bands from different types of defects overlap and may even have the same positions in the PL spectrum. Then, the PL bands can only be distinguished and resolved through advanced analysis of their shape and PL decay in steady-state and time-resolved PL measurements and by comparing the capture coefficients.

In this work, we apply PL methods to find parameters for several defect-related PL bands in GaN grown by HVPE. The coefficient *C*_*nA*_ is determined for the ultraviolet luminescence (UVL), blue luminescence (BL1), green luminescence (GL1), yellow luminescence (YL1), and red luminescence (RL1) bands. We add the number 1 to the commonly used abbreviations to identify a defect-related band with specific properties, because more than one defect can cause red, yellow, green, and blue bands in undoped GaN. The calculated concentrations of free electrons at selected temperatures in several samples are compared with values obtained from temperature-dependent Hall-effect measurements. The knowledge of these capture coefficients allows us to find the concentration of free electrons in GaN; in particular, in regions not accessible by other techniques, such as the region close to the GaN/sapphire interface.

## Results

### Temperature-dependent Hall-effect data

**Figure 1:**
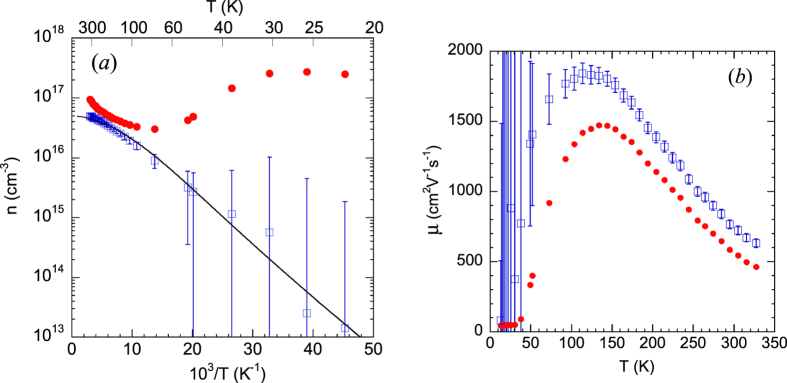
Temperature-dependent Hall-effect data. HVPE GaN (sample 2057). The measured values (solid circles) were corrected using a two-layer model (empty squares) with *μ* = (44.5 ± 5.0) cm^2^/Vs and *n*_□_/*d* = (2.72 ± 0.25) × 10^17^ cm^−3^ for the degenerate interface layer, where n_□_ is the sheet electron concentration and *d* = 24 μm is the total GaN thickness. The solid line is fit using *N*_*D*_ = 1 × 10^17^ cm^−3^, *N*_*A*_ = 5 × 10^16^ cm^−3^, and *E*_*D*_ = 14 meV. The error bars reflect the uncertainty in *μ* and *n* in the degenerate layer estimated from the low-temperature data where the bulk conductivity is negligible. **(a)** The concentration of free electrons *n*, **(b)** the electron mobility *μ*.

Figure 1 shows the temperature dependence of the concentration of free electrons *n* and their mobility *μ* for one of the undoped GaN samples. The as-measured dependence for the concentration of free electrons (solid circles) clearly indicates the presence of a degenerate layer (presumably near the GaN/sapphire interface), which moderately affects the measured *n* and *μ* at temperatures above 100 K and completely dominates in the conductance at lower temperatures. The two-layer model^12^ was used to determine the values of *n* and *μ* in the bulk part of the GaN layer and their temperature dependence. The restored temperature dependence of *n* (empty squares in Fig. 1a) can be fit with the following expression^12^


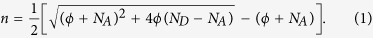


here, *N*_*D*_ and *N*_*A*_ are the concentrations of shallow donors and all acceptors, respectively, 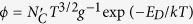, where *g* is the degeneracy of the donor state (*g* = 2), 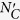 is the effective density of states in the conduction band at *T* = 1 K (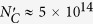 cm^−3^ for m* = 0.22 *m*_0_), *k* is Boltzmann’s constant, and *E*_*D*_ is the donor activation energy. The exact values of *N*_*D*_ and *N*_*A*_ are not important for this work, and we estimate *N*_*A*_ as 5 × 10^16^ cm^−3^ for this sample because the temperature dependence of electron mobility in Fig. 1b is nearly identical to the one in ref. 12, where *N*_*A*_ has been determined from the detailed analysis of the *μ(T)* dependence. Then, from the best fit, shown with a solid line in Fig. 1b, we find *N*_*D*_ = 1 × 10^17^ cm^−3^ and *E*_*D*_ = 14 meV, parameters which are very similar to those found in ref. 12. We have conducted the temperature-dependent Hall-effect measurements and found the *n(T*) dependences for all of the GaN samples where the degenerate interfacial layer could affect the conductivity (Table 1). The obtained concentrations of free electrons for the bulk part of GaN were used for the quantitative analysis of PL data, which were obtained from the front side of the samples, and far from the degenerate interfacial layer.

### Steady-state and time-resolved PL spectra

**Figure 2:**
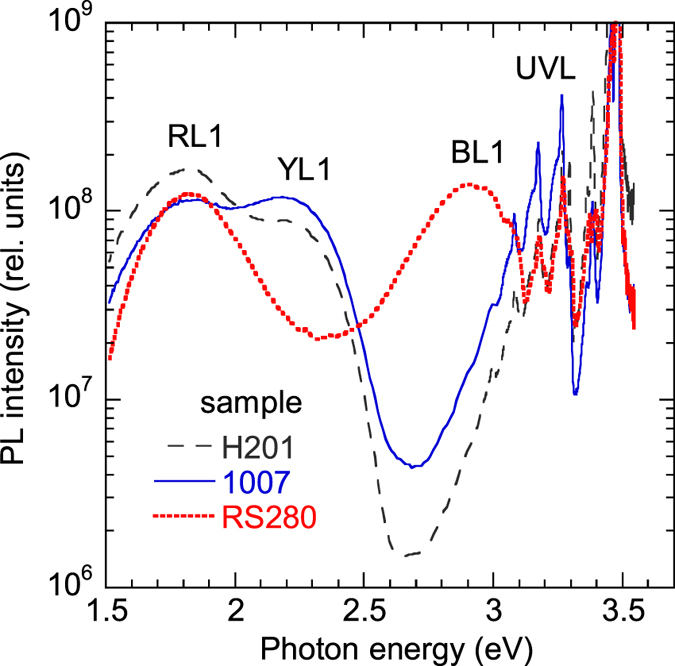
Steady-state PL spectra from undoped GaN. *T* = 18 K and *P*_*exc*_ = 10^−3^ W/cm^2^. The upper part of the exciton emission is not shown.

Typical steady-state PL spectra at low temperature from the studied GaN samples are shown in Fig. 2. A red band with a maximum at 1.8 eV is often observed in HVPE-grown GaN^10^. Due to the fact that there are several different defects responsible for the red luminescence in GaN, careful measurements must be performed to determine which band is present in a given sample. The RL1 band with a maximum at 1.8 eV is often observed in the PL spectrum from HVPE GaN samples and does not exhibit any fine structure. The decay of the RL1 band is nonexponential at 15 K, which is typical for donor-acceptor-pair (DAP) recombination^15^. With increasing temperature, the PL decay approaches an exponential dependence:


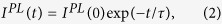


due to the gradual replacement of DAP-type recombination with transitions of electrons from the conduction band to the acceptor level. The characteristic PL lifetime *τ* for the RL1 band at temperatures between 100 and 300 K is very long (usually in the millisecond range)^10^.

In high-resistivity GaN grown in Ga-rich conditions, another red band can be observed with a maximum at about 1.8 eV. We previously labeled it the RL2 band and attribute it to an internal transition within some unknown defect^10^. The exact type of the transition is unknown, but the proof that it is internal (i.e., between two energy levels of the same defect) follows from the fact that the PL decay of the RL2 band is exponential at very low temperature (15 K) and the PL lifetime is independent of the concentration of free electrons. The characteristic feature of the RL2 band is that its PL lifetime decreases from 110 to 2 μs with increasing temperature from 15 to 100 K^10^.

A third red band, called the RL3 band hereafter, is observed in some HVPE GaN samples, and was previously reported to have fine structure with the zero-phonon line (ZPL) at 2.36 eV^16^. Time-resolved PL measurements have since shown that the fine structure should be attributed to a yellow luminescence band often covered by the RL3 band in the PL spectra. Additionally, time-resolved PL data show that the RL3 band (analyzed in ref. 16) has an exponential and very fast decay after a laser pulse. The characteristic PL lifetime for the RL3 band is about 15 ns at temperatures between 15 and 200 K. Such exponential, temperature-independent PL decay indicates that an internal transition is responsible for this PL band.

Both the RL1 and RL3 bands can be observed in HVPE-grown *n*-type GaN with a free-electron concentration of about 10^17^ cm^−3^. We can clearly distinguish the RL1 and RL3 bands through their time-resolved PL behavior, but currently we are unable to find the reason why one or another red band appears in different GaN samples grown by HVPE in apparently similar conditions. In this work, we will limit our interest to the slow RL1 band, because it is caused by electron transitions from shallow donors (at low temperature) and from the conduction band (at elevated temperatures) to a deep defect level. Detailed studies of the RL3 band will be reported elsewhere.

A yellow luminescence (YL) band with a maximum at about 2.1–2.2 eV in undoped GaN may also be caused by several different defects. It may be difficult to distinguish the YL bands related to different defects, especially in samples where other PL bands are strong and the overlap with the YL band is significant. One of the YL bands, labeled the YL1 band, has a ZPL at 2.57 eV and band maximum at 2.20 eV at low temperature^17^. This band was observed in several HVPE-grown GaN, as well as in undoped, carbon-containing GaN and Si-doped GaN layers grown by metal-organic chemical vapor deposition (MOCVD).

In samples with a strong RL3 band, another YL band can be resolved in time-resolved PL measurements. It has a maximum at 2.10 eV and fine structure on its high energy side with the ZPL at 2.36 eV. Our preliminary results indicate that this yellow band is most probably caused by the same defect as the RL3 band. This band will be called the YL3 band, hereafter, and details of its study will be reported elsewhere. It is likely that YL bands other than YL1 and YL3 can be found in GaN. However, in this work, only samples with the YL band reliably identified as YL1 are included in the detailed analysis. For example, the steady-state and time-resolved PL spectra from sample RS280 contain red and yellow bands. These bands (apparently the RL3 and YL3 bands) have properties different from those of the RL1 and YL1 bands and therefore are excluded from the analysis.

In some HVPE GaN samples, a blue luminescence band with a maximum at 2.9 eV and the characteristic fine structure at the high-energy side can be well resolved in the low-temperature PL spectrum (samples RS280, 1601, T1011, T2015, and B73). This band is associated with Zn_Ga_ acceptors unintentionally introduced during GaN growth with HVPE or MOCVD^10^. The band will be labeled BL1 to distinguish it from the BL2 band with a maximum at 3.0 eV presumably caused by carbon-hydrogen complexes^18^. In some samples, the BL1 band is obscured by a UVL band at *T* < 120 K but can be observed at temperatures between 150 and 200 K where the UVL band is quenched (samples 104, 201, 202, 203). The thermal quenching of the BL1 band with an activation energy of about 350 meV begins at *T* > 180 K. In some samples no BL1 band could be detected at any temperature (samples 3, 2057, cvd3533, cvd3540).

A UVL band with a main peak at 3.26 eV was observed in the low-temperature PL spectra of almost all the studied GaN samples, yet its intensity varied within a wide range (two orders of magnitude). The UVL band is attributed to transitions from shallow donors (at low temperature) or from the conduction band (at elevated temperatures) to a shallow acceptor with the ionization energy of 0.2 eV^10^. It is likely that the shallow acceptor is the Mg_Ga_, and Mg is unintentionally introduced in low concentrations during sample growth.

With increasing temperature, thermal quenching of the UVL band begins at *T* > 100 K and the band completely disappears by *T* = 200 K. Quenching of the UVL and BL1 bands in *n*-type GaN is caused by thermal emission of holes from a shallow acceptor (for the UVL band) and from the Zn_Ga_-related acceptor (for the BL1 band) to the valence band. The intensities of the YL1 and RL1 bands are nearly constant up to room temperature.

The shape of a broad, defect-related PL band, *I*^*PL*^(*ħω*), can be simulated with the following formula derived from a one-dimensional configuration coordinate model^19^


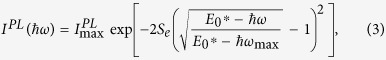


where 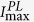 is the intensity of the PL band maximum, *S*_*e*_ is the Huang-Rhys factor for the excited state, *ħω* and *ħω*_max_ are the photon energy and position of the band maximum, 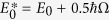, *E*_0_ is the ZPL energy, and *ħ*Ω is the energy of the dominant phonon mode in the excited state. Table 2 presents the parameters for different PL bands obtained from analysis of the PL band shapes in a large number of GaN samples grown by MOCVD and HVPE. In addition to the PL bands analyzed in this work, it includes also the parameters for the RL2, GL2 and BL2 bands which are observed only in high-resistivity GaN^10^. We use these parameters to resolve PL bands in steady-state and time-resolved PL spectra when the overlap between bands is significant.

**Figure 3:**
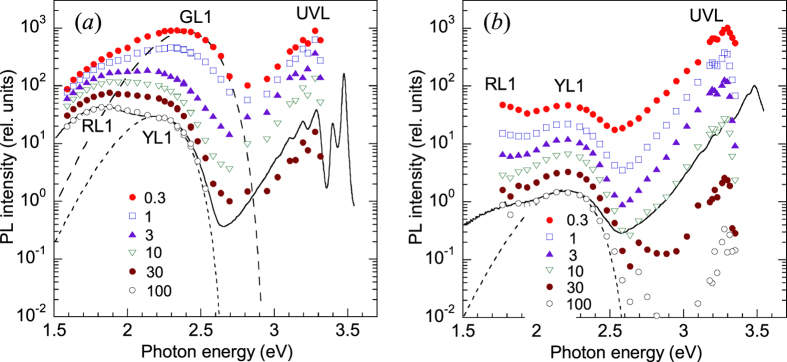
Time-resolved PL spectra at *T* = 100 K for sample 201. **(a)** PL excited from the front side. **(b)** PL excited from the backside (through the sapphire substrate). The points are the PL intensity at indicated time delays (in μs) after the laser pulse. The solid line is the steady-state PL spectrum at *P*_*exc*_ = 1 mW/cm^2^ (arbitrarily shifted along the vertical axis for comparison with the time-resolved PL spectrum at 100 μs). The dashed and dotted lines are calculated using Eq. (3) with the following parameters: *S*_*e*_ = 8.5, *E*_0_^*^ = 2.93 eV, *ħω*_max_ = 2.40 eV (for the GL1 band) and *S*_*e*_ = 7.4, *E*_0_^*^ = 2.66 eV, *ħω*_max_ = 2.20 eV (for the YL1 band).

In time-resolved PL measurements, defect-related PL bands can often be easily resolved by comparing PL spectra obtained at different time delays after the pulse excitation, because different PL bands have different characteristic lifetimes. Examples of time-resolved PL spectra at time delays between 0.3 and 100 μs for two sides of the same sample are given in Fig. 3.

Time-resolved PL spectra are sometimes able to reveal defect-related PL bands that are difficult to resolve in steady-state PL spectra where they may be obstructed by other PL bands. The deconvolution of defect-related PL bands in time-resolved PL spectra is simple and reliable when the characteristic PL lifetimes and their temperature dependences for the overlapped PL bands are very different. A good example is a green luminescence band (GL1, also known as GL) which has a maximum at 2.4 eV in HVPE-grown GaN (Fig. 3a). The GL1 band is usually not visible in the steady-state PL spectrum (Fig. 2); however, it can be observed in time-resolved PL measurements at short time delays (Fig. 3a) and can be fit using Eq. (3) with parameters previously found for the GL1 band in samples where the GL1 band is much stronger and the overlap with other PL bands is negligible^6^. This PL band, commonly observed in conductive *n*-type GaN, has an exponential decay after a laser pulse, with a characteristic PL lifetime of about 1–2 μs at temperatures between 15 and 100 K. The PL lifetime for this band increases at temperatures above 100 K, which makes this PL band different from all other defect-related PL bands in GaN^10^. The GL1 band is preliminarily attributed to an internal transition from an excited state to the ground state of the 0/+ transition level of the isolated C_N_ defect^6^,^20^. We call this band the GL1 band to distinguish it from the GL2 band with a maximum at 2.36 eV observed in high-resistivity GaN and attributed to V_N_^19^.

**Figure 4:**
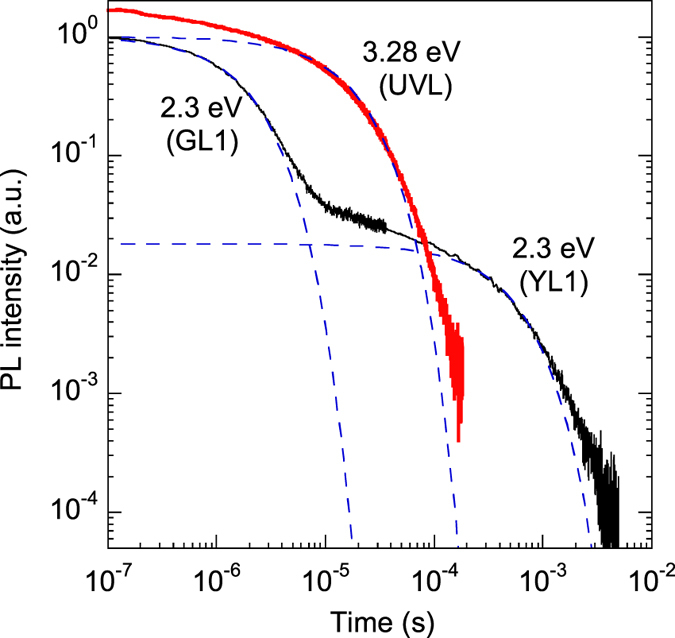
Decay of PL after a laser pulse. *T* = 100 K, sample 1007. The dashed lines are calculated using Eq. (2) with *τ* = 1.7 × 10^−5^, 1.8 × 10^−6^, and 4.8 × 10^−4^ s for the UVL, GL1, and YL1 bands, respectively.

At short time delays, the UVL and GL1 bands often dominate in the PL spectrum (Fig. 3a), because they have the shortest lifetimes (about 10 and 1 μs, respectively, at 100 K). At longer time delays, the UVL and GL1 bands disappear, and the YL1 and RL1 bands become dominant, with PL lifetimes exceeding 100 μs. As an example, Fig. 4 shows PL decays at 3.28 eV (the UVL band maximum) and at 2.3 eV (close to the maxima of the YL1 and GL1 bands) at 100 K.

### Temperature dependence of PL lifetime

Temperature dependences of PL intensity and PL lifetime for defect-related PL bands can typically be described with the following expressions^14^:


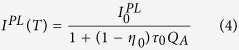


and


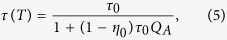


respectively, where


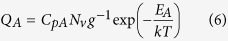


and


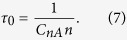


Here, *C*_*nA*_ and *C*_*pA*_ are the electron- and hole-capture coefficients, respectively, for a defect *A* (usually an acceptor) with ionization energy *E*_*A*_ for bound holes, *N*_*v*_ is the effective density of states in the valence band, and 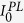, *τ*_0_, and *η*_0_ are the PL intensity, PL lifetime, and the absolute internal quantum efficiency of PL from defect *A*, respectively, at low temperatures when the thermal quenching of PL can be ignored. According to Eq. (4), the defect-related PL intensity is constant at temperatures below *T*_0_ and decreases with increasing temperature at temperatures above *T*_0_, where *T*_0_ can be found from the condition (1 − *η*_0_)*τ*_0_*Q*_*A*_ = 1. The temperature-dependent portion of the *I*^*PL*^(*T*) dependence is called the PL quenching. The PL lifetime changes with temperature in a very similar way [compare Eqs (4) and (5)], with the difference that at *T* < *T*_0_ the PL lifetime *τ* = *τ*_0_ is expected to decrease in nondegenerate samples [Eq. (7)] due to the temperature dependence of *n*, which is given by Eq. (1).

**Figure 5:**
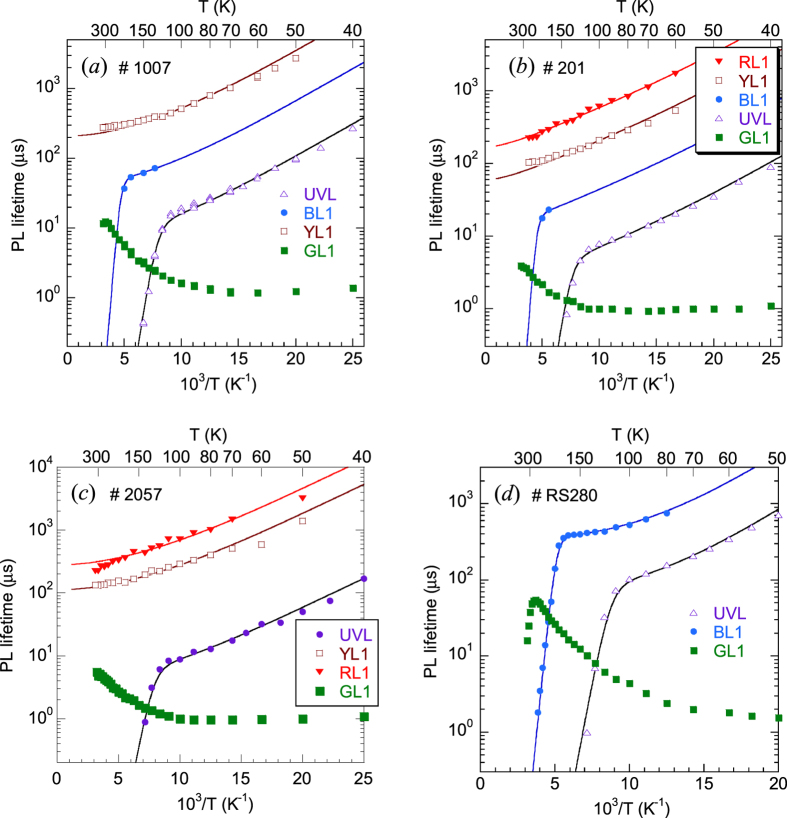
Temperature dependence of PL lifetime for the main PL bands in GaN. **(a)** sample 1007, **(b)** sample 201, **(c)** sample 1007, **(d)** sample RS280. The solid lines are calculated using Eqs (1) and (5) with the following parameters: *E*_*D*_ = 14 meV for samples 1007, 2057, and 201, and *E*_*D*_ = 20 meV for sample RS280; *E*_*A*_ = 186 meV for the UVL band and *E*_*A*_ = 350 meV for the BL1 band (all samples); *C*_*pA*_ = 10^−6^ cm^3^/s for the UVL band and *C*_*pA*_ = 7 × 10^−7^ cm^3^/s for the BL1 band (all samples); *N*_*D*_ = 1.1 × 10^17^ cm^−3^ (sample 1007), 1 × 10^17^ cm^−3^ (sample 2057), 3 × 10^17^ cm^−3^ (201), and 1.6 × 10^16^ cm^−3^ (RS280); *N*_*A*_ = 5 × 10^16^ cm^−3^ (samples 1007, 2057, and 201) and 1.0 × 10^16^ cm^−3^ (RS280); *C*_*nA*_ = 2.6 × 10^−12^, 5.6 × 10^−12^, 2 × 10^−12^, and 2 × 10^−12^ cm^3^/s for the UVL band in samples 1007, 2057, H201, and RS280, respectively; *C*_*nA*_ = 4.2 × 10^−13^, 3.2 × 10^−13^, and 3.7 × 10^−13^ cm^3^/s for the BL1 band in samples 1007, 201, and RS280, respectively; *C*_*nA*_ = 8 × 10^−14^, 1.7 × 10^−13^, 6.8 × 10^−14^ cm^3^/s for the YL1 band in samples 1007, 2057, and 201, respectively; and *C*_*nA*_ = 7.4 × 10^−14^ and 2.4 × 10^−14^ cm^3^/s for the RL1 band in samples 2057 and 201, respectively.

Figure 5 shows temperature dependences of the PL lifetime for defect-related PL bands in selected samples. The temperature behavior of the GL1 lifetime is discussed separately^20^. As for other PL bands, the experimentally found *τ(T*) dependences can be fit with Eq. (5), with the assumption that the parameter *C*_*nA*_ is independent of temperature. The temperature dependences of *n* for samples 1007, 2057, and 201 were found from the temperature-dependent Hall effect by using the two-layer model^12^, similarly to an example shown in Fig. 1. For sample RS280, the two-layer model produced unreasonable parameters (such as *E*_*D*_ = 45 meV), and the *n(T*) dependence was reconstructed based on the experimentally measured values of *τ*_0_.

At *T* < 40 K, the PL decay after a laser pulse for the UVL, BL1, YL1, and RL1 bands is nonexponential and close to the *t*^−1^ dependence. This is typical for DAP recombination, where transitions from shallow donors to defects responsible for the above PL bands dominate^15^. At *T* > 40 K, transitions from the conduction band to the same defect levels become dominant, and the PL decay becomes nearly exponential, so that the characteristic PL lifetime can be found according to a definition proposed in ref. 21. The PL quenching was observed for the UVL and BL1bands, but was not observed for the YL1 and RL1 bands up to room temperature, because the defects responsible for the YL1 and RL1 bands have larger ionization energies.

**Figure 6:**
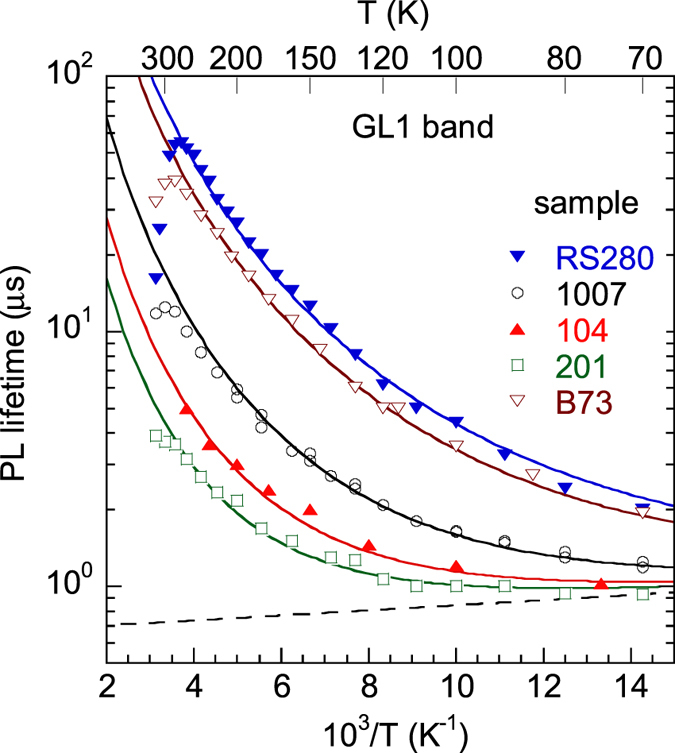
Temperature dependence of the PL lifetime for the GL1 band in HVPE GaN. The dashed line is *τ*_2_ given with Eq. (8). The solid lines are calculated using Eq. (10), with *τ*_1_ and *τ*_2_ given by Eqs (9) and (8) with *τ*_20_ = 0.67 μs and *E*_2_ = 2 meV and *a* = 2.8. Parameter *τ*_1_(100 K) is sample-dependent: 3.5 μs (sample RS280), 2.6 μs (B73), 0.75 μs (1007), 0.3 μs (104), and 0.17 μs (201).

The temperature dependence of *τ* for the GL1 band is unusual (Figs 5 and 6). At low temperature (*T* ≈ 20–50 K), the PL decay is exponential, and the PL lifetime is about the same for samples with different concentrations of free electrons. In this temperature region, the PL lifetime slowly decreases with increasing temperature and can be fit with the following empirical expression^20^ (dashed line in Fig. 6):


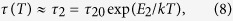


where *τ*_20_ = 0.67 μs and *E*_2_ = 2 meV. At higher temperatures (*T* ≈ 100–280 K), the PL lifetime can be described with the following expression:


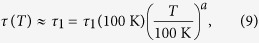


where the parameter *τ*_1_(100 K) is inversely proportional to the concentration of free electrons, i.e. sample-dependent, and *a* ≈ 2.8. For a wide temperature range (from 20 to 280 K), *τ* for the GL1 band represents the sum of *τ*_1_ and *τ*_2_:


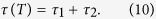


The solid lines in Fig. 6 are the fits using Eq. (10) with parameters indicated in the figure caption.

### Determination of the electron capture coefficients

**Figure 7:**
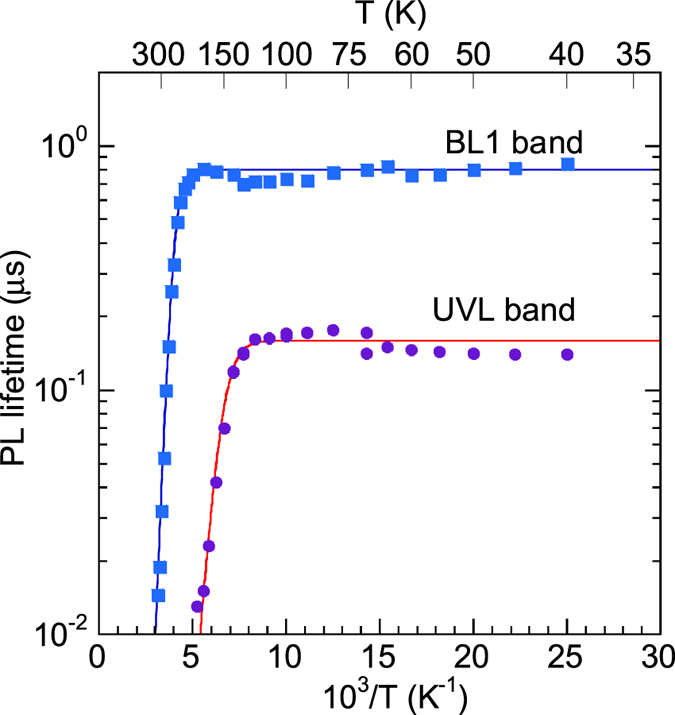
Temperature dependence of PL lifetime for PL bands in a degenerate Si-doped GaN. The solid lines are calculated using Eq. (5) with the following parameters: *E*_*A*_ = 170 meV for the UVL band and *E*_*A*_ = 320 meV for the BL1 band; *C*_*pA*_ = 10^−6^ cm^3^/s for the UVL band and *C*_*pA*_ = 7 × 10^−7^ cm^3^/s for the BL1 band; *τ*_0_ = 0.16 and 0.8 μs for the UVL and BL1 bands, respectively. Sample 1601.

The electron-capture coefficient *C*_*nA*_ can be found from Eq. (7) when *n* and *τ*_0_ are known. From good agreement between the experimentally found temperature dependences of PL lifetimes and *τ*_0_ calculated from Eq. (7) with the assumption that *C*_*nA*_ is constant (Fig. 5), we conclude that *C*_*nA*_ for the studied PL bands (except for the GL1 band) has no or almost no temperature dependence for the range of temperatures used. Additional proof that *C*_*nA*_ is independent of temperature can be obtained from data for degenerate GaN samples, where *n* is independent of temperature (Fig. 7).

To find the absolute values of *C*_*nA*_, we selected 14 HVPE-grown GaN samples for which the temperature dependence of the concentration of free electrons was reliably determined (samples in Table 1 excluding samples with prefixes RS and cvd). The UVL band was present in PL spectra of all these samples, and its electron-capture coefficient could be reliably determined as *C*_*n*,*UVL*_ = (3.2 ± 0.3) × 10^−12^ cm^3^/s at 100 K. The average values of *C*_*nA*_ and the standard deviations of the mean for other PL bands at selected temperatures were determined from smaller sample sets (4–10 samples) and are shown in Table 3.

The above-determined coefficients *C*_*nA*_ may contain a systematic error if GaN layers are not uniform. Indeed, the Hall effect method, even after applying the two-layer model, gives the average value of *n* in bulk part of a layer with the thickness of several micrometers, whereas the concentration of free electrons calculated from time-resolved PL measurements corresponds to a thin layer (200–400 nm) near the sample surface. However, sometimes, the relative values of coefficients *C*_*nA*_ for different PL bands are more important, and they can be found with higher accuracy as will be shown below.

According to Eq. (7), *relative* capture coefficients for different PL bands can be determined from the ratio of the PL lifetimes for a given sample, even if the concentration of free electrons is unknown or the layer is not uniform. The matter is that PL lifetimes for all defect-related bands correspond to the same region of the sample from which PL signal is collected. To improve statistics, we included in analysis 12 more GaN samples grown by HVPE, for which the concentration of free electrons was not analyzed with the temperature-dependent Hall effect or was questioned (such as sample RS280). Since no temperature dependence of *C*_*nA*_ was noticed for the UVL, BL1, YL1, and RL1 bands, and the most suitable temperature where all these bands are observed and where Eq. (5) reduces to *τ(T*) ≈ *τ*_0_ = (*C*_*nA*_*n*)^−1^ is *T* ≈ 100 K (Fig. 5), the relative PL lifetimes were calculated for this temperature (Table 4). The values of *C*_*nA*_ calculated from the ratios of PL lifetimes, by using *C*_*n*,*UVL*_ = (3.2 ± 0.3) × 10^−12^ cm^3^/s, are also given in Table 4.

For the GL1 band, the value of *τ* at 100 K cannot be used to determine the concentration of free electrons because it corresponds to an internal transition at this temperature^20^. The value of *τ* at 250 K is the most suitable parameter of GL1, because for *T* < 250 K, the low-temperature component of the lifetime *τ*_2_, which is independent of *n*, may have a significant contribution to *τ* in samples with a relatively high concentration of free electrons. On the other hand, the thermal quenching of the GL1 band may begin at *T* > 250 K for samples with low concentrations of free electrons (Fig. 6). From the time-resolved PL and temperature-dependent Hall effect data for 10 samples, we find that *C*_*n*,*GL*1_ = (3.7 ± 0.5) × 10^−12^ cm^3^/s for the GL1 band at 250 K. Independently, for a larger set of samples (19 samples) where both the UVL and GL1 bands were observed, we find that *τ*_*GL1*_(250 K)/*τ*_*UVL*_(100 K) = 0.466 ± 0.024.

### PL from GaN/sapphire interface

**Figure 8:**
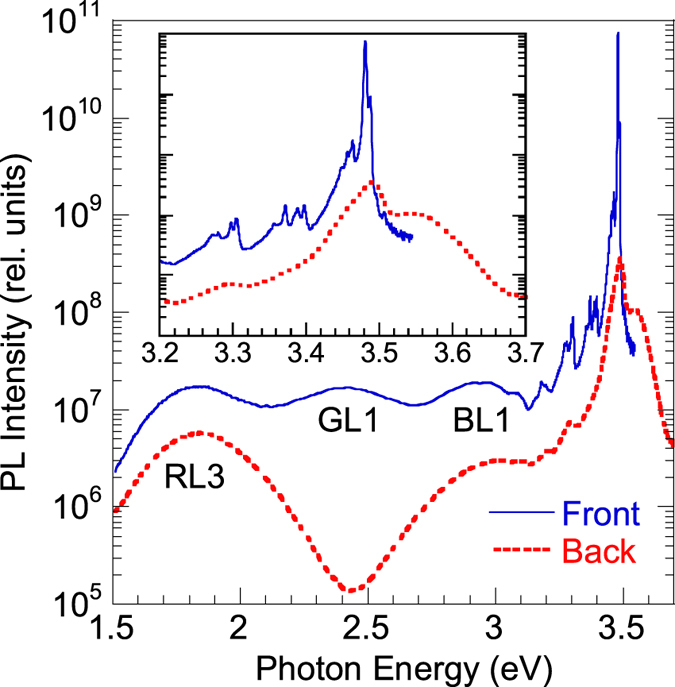
Steady-state PL spectrum from the front side and backside (the GaN/sapphire interface region) of sample RS280. The spectra were measured in identical conditions at 18 K and with *P*_*exc*_ = 0.03 W/cm^2^. The inset shows the near-band-edge emission with better resolution.

To confirm our assumption that the concentration of free electrons determined from the room-temperature Hall effect measurements is overestimated due to a conductive shunting layer near the GaN/sapphire interface, we studied PL from the sapphire side for several HVPE GaN samples. In these samples, the steady-state PL indicates the presence of a degenerate GaN layer. An example of the steady-state PL spectrum from the backside of a GaN layer is shown in Fig. 8. As is expected for a degenerate GaN layer, the near-band-edge emission is broad and extends up to ~3.7 eV. In regards to the defect-related PL bands, the GL1 band is missing in the PL spectrum from the backside. This agrees with our observations, according to which the GL1 band can be found only in high-purity GaN. Similar results were obtained for other samples (including sample 201 analyzed in Fig. 3).

In time-resolved PL measurements, the decay of the defect-related PL intensity from the backside is fast and nonexponential even at 100 K (Fig. 3b), so that the characteristic PL lifetime cannot be determined. The fast component of the PL decay apparently originates from the degenerate GaN layer near the GaN/sapphire interface. The PL decay is nonexponential, because deeper layers with gradually improving quality and decreasing concentration of free electrons also contribute to the PL. As in the steady-state PL spectrum, the GL1 band is not observed in the time-resolved PL spectra (Fig. 3a). However, other PL bands, such as the UVL, YL1, and RL1 bands, can be resolved.

## Discussion

We have established that the electron-capture coefficients for the UVL and BL1 bands in nondegenerate and degenerate GaN are independent of temperature from 40 to 100 K (the UVL band) and from 40 to 180 K (the BL1 band). For the YL1 and RL1 bands, the typical range where *C*_*nA*_ is independent of temperature is from 50 to 300 K. For some samples, a weak temperature dependence was observed in this range, but it can be caused by errors in the determination of the free electron concentration from the Hall effect. The independence of the electron-capture coefficients with temperature agrees with theoretical predictions for radiative transitions^22^.

In sharp contrast to the above behavior, the coefficient *C*_*nA*_ determined for the GL1 band from Eq. (7) has a strong temperature dependence; namely, *C*_*nA*_ is inversely proportional to the cube of the temperature. The observation of the temperature dependence of *C*_*nA*_ is a very interesting phenomenon, and we are not aware of similar behavior for other PL bands in GaN or other semiconductors. We have explained it recently using a two-step recombination model^20^. In the first step of the electron-hole recombination, an electron is captured by an excited Coulomb state of a positively charged defect (presumably 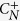), and this nonradiative capture is a classic case of capture by a giant trap. In the second step, the captured electron recombines with one of the two holes trapped at the defect. This is an internal radiative transition, which produces the GL1 band. According to this model, the measured PL lifetime in time-resolved PL experiments is approximately equal to the sum of the nonradiative and radiative stages of the recombination.

It is important for practical purposes that the PL lifetime for the GL1 band is related to the concentration of free electrons at temperatures where it is governed by the slower nonradiative capture of free electrons by an excited state (e.g., at 250 K). Thus, by measuring *τ*_0_ for the GL1 band at 250 K we can determine *n* at this temperature by using Eq. (7) with *C*_*n*,*GL*1_ = 3.7 × 10^−12^ cm^3^/s. At room temperature (293 K), *C*_*n,GL1*_ ≈ 2 × 10^−12^ cm^3^/s, yet this value is less reliable than the one at 250 K, especially for samples with very low concentrations of free electrons, because the PL quenching begins at 280 K in these samples (Fig. 6). This finding may be very useful for a contactless evaluation of the free electron concentration in high-quality, thick GaN grown by HVPE, where the GL1 band is often the only defect-related PL band at room temperature.

After quantifying the capture coefficients, *n* and its temperature dependence can be found from the PL lifetime of the UVL, YL1, GL1, or RL1 bands if they are present in a PL spectrum. A good example is sample RS280, which is a 27-μm-thick undoped GaN layer on a sapphire substrate. From Hall-effect measurements, the concentration of free electrons at *T* = 250–293 K was found to be about 1 × 10^18^ cm^−3^. The attempts to find *n* in the bulk part of the layer by using the two-layer model were not successful (unrealistic change of *n* from 2.9 × 10^17^ to 1.3 × 10^16^ cm^−3^ with decreasing temperature from 250 to 100 K), probably because of a very large contribution of the degenerate interface layer to the total electrical conductivity. However, the time-resolved PL measurements indicated that the sample has the lowest concentration of free electrons among all the studied samples, because the PL lifetimes were the longest for each PL band (Table 1). By using the electron-capture coefficients for the UVL, BL1, and GL1 bands, we have estimated that *n* = (3.0 ± 0.2) × 10^15^ cm^−3^ at *T* = 100 K and *n* = (6 ± 1) × 10^15^ cm^−3^ at *T* = 180–250 K in this sample, at least in the region from which the PL signal is collected (0.2–0.4 μm-thick layer near the sample surface).

Defects causing PL signals can be distinguished by characteristic properties of related PL bands, even when the chemical origins and structures of defects are not known. In majority of publications, a PL band is recognized by only its position in the PL spectrum. However, the shape and position of a broad PL band can be affected by contributions from other PL bands, as well as they may be distorted by the measurement system if appropriate corrections for spectral response of the system are not applied. Among other characteristic parameters, the electron- and hole-capture coefficients can be easily determined and serve as fingerprints of defects.

It is safe to expect that PL bands with the same positions, shapes and capture coefficients belong to the same defect. For example, the UVL and BL1 bands are caused by an unknown shallow acceptor and the Zn_Ga_ acceptor in all studied GaN samples. The PL lifetime of the UVL band could be reliably measured at 100 K in 26 undoped and Si-doped HVPE-grown GaN samples. In 19 samples from this set, the GL1 band could be observed in time-resolved PL measurements at 250 K. The ratio of the GL1 lifetime to the UVL lifetime was nearly the same in all the samples (between 0.36 and 0.77), with the average value of 0.47 and a statistical error of 5%. Even more reproducible ratios were observed for the BL1 and UVL bands at 100 K (the average of 4.7 with a statistical error of just 2.5%) in the samples where the PL lifetime of the BL1 band could be reliably measured. We conclude that the UVL, BL1, and GL1 bands have the same origins in all these samples.

In contrast to the reproducible data for the UVL, BL1, and GL1 bands, the data for the YL band in the set of 26 samples divided into two groups, and we were able to distinguish two bands with different properties. The YL1 band was observed in 10 samples and the YL3 band was observed in 11 other samples. The YL1 band is the commonly observed YL band, with a maximum at 2.2 eV and with the ratio of its lifetime to the UVL lifetime equal to 29.8 ± 1.2 at 100 K. In 8 out of the 10 samples, the ZPL of the YL1 band was observed at 2.57 eV at 18 K^20^, which serves as an additional fingerprint.

However, in 11 other samples the RL3 band with a maximum at 1.8 eV was the dominant PL band in this spectral region. In time-resolved measurements, the RL3 band decayed very fast (with the lifetime shorter than 20 ns), and the YL3 band with a maximum at 2.10 eV evolved with time delay. Our preliminary results show that the ZPL at 2.36 eV belongs to the YL3 band (and not to the red band as was reported earlier^16^), and the YL3 and RL3 bands are most likely caused by the same defect. The preliminary results are complicated, and detailed study of the YL3 and RL3 bands in HVPE-grown undoped GaN will be reported elsewhere. It is important to mention here that the YL1 and YL3 bands have different coefficients *C*_*nA*_. We determined for 5 samples with known (from the Hall effect) concentrations of free electrons at 100 K that *C*_*nA*_ = (2.0 ± 0.5) × 10^−13^ cm^3^/s for the YL3 band, as opposed to *C*_*nA*_ = (1.1 ± 0.2) × 10^−13^ cm^3^/s for the YL1 band. For a larger set of 11 samples where the YL3 and UVL bands were detected, the ratio of their lifetimes was found to be 18.3 ± 1.7 at 100 K, well outside of the statistical range for the YL1 band (29.8 ± 1.2).

In our Si-doped GaN grown by MOCVD, only the UVL and YL1 bands were observed, with parameters nearly identical to those for HVPE-grown GaN. Previously, the values of *C*_*nA*_ for the UVL, BL1, and YL1 bands in undoped GaN grown by MOCVD were reported to be 4 × 10^−12^, 4 × 10^−13^, and 7 × 10^−14^ cm^3^/s^10^,^21^, not much different from the current study. The GL1 and RL1 bands were not observed in GaN grown by MOCVD.

In summary, new classification of the defect-related PL bands in undoped GaN is suggested to distinguish bands with similar position (color) but different origin caused by different defects. The electron-capture coefficients for the UVL, BL1, GL1, YL1, and RL1 bands in undoped GaN grown by HVPE are determined based on the statistical analysis of many undoped and Si-doped GaN samples. These coefficients can be used to determine the concentration of free electrons in GaN from time-resolved PL measurements. The method is especially useful when a GaN sample is not uniform and contains layers with higher electrical conductivity, which may shunt the bulk of the sample. It can also be used for GaN-based devices or structures containing buried quantum wells or cap layers. In high-purity GaN samples grown by HVPE, the GL1 band with a maximum at 2.4 eV is often the only defect-related PL band, which can be reliably identified due to its very special properties. The PL lifetime for this PL band at temperatures close to room temperature can be used to determine the concentration of free electrons in GaN. The ratios of PL lifetimes between different PL bands can be used for a reliable identification of the PL bands when more than one defect causes PL bands with similar shapes and positions.

## Methods

### Samples

We investigated more than 20 samples, 3–30 μm-thick, unintentionally doped and Si-doped GaN layers grown by HVPE on *c*-plane sapphire substrates. For several samples from this set, the temperature-dependent Hall effect was investigated (first 12 samples in Table 1). Sample 1601 is degenerate due to doping with Si. Two other Si-doped samples (cvd3533 and cvd3540) were grown by MOCVD. We also included in the analysis two samples grown by HVPE at TDI, Inc. (T1011 is undoped GaN, and T2015 is Si-doped GaN), and a 200-μm-thick, freestanding undoped GaN template grown by HVPE at the Samsung Advanced Institute of Technology (sample B73). The concentration of free electrons in these samples was also determined by analyzing the temperature-dependent Hall effect measurements.

### Experimental details

Steady-state PL was excited with an unfocused He-Cd laser (30 mW, 325 nm), dispersed by a 1200 rules/mm grating in a 0.3 m monochromator and detected by a cooled photomultiplier tube. Calibrated neutral-density filters were used to attenuate the excitation power density (*P*_exc_) over the range 10^−5^–0.1 W/cm^2^. Time-resolved PL was excited with a pulsed nitrogen laser (pulses with duration of 1 ns and repetition frequency of 6 Hz, and photon energy of 3.68 eV) and analyzed with an oscilloscope. A closed-cycle optical cryostat was used for temperatures between 15 and 320 K. The absolute internal quantum efficiency of PL, *η*, is defined as *η* = *I*^*PL*^/*G*, where *I*^*PL*^ is the integrated PL intensity from a particular PL band and *G* is the concentration of electron-hole pairs created by the laser per second in the same volume. To find *η* for a particular PL band, we compared its integrated intensity with the PL intensity obtained from a calibrated GaN sample^23^,^24^. All of the samples were studied under identical conditions.

## Additional Information

**How to cite this article**: Reshchikov, M. A. *et al*. Determination of the electron-capture coefficients and the concentration of free electrons in GaN from time-resolved photoluminescence. *Sci. Rep.* **6**, 37511; doi: 10.1038/srep37511 (2016).

**Publisher's note:** Springer Nature remains neutral with regard to jurisdictional claims in published maps and institutional affiliations.

## Figures and Tables

**Table 1:** Parameters of the samples and the PL lifetime for main PL bands in GaN.

Sample number	Thickness (μm)				*τ* (μs)				
		*n* (10^16^ cm^−3^)			UVL	BL1	YL1	RL1	GL1
		100 K	180 K	250 K	100 K	180 K	180 K	180 K	250 K
3	7	2.2	5	6.4	15		250	600	6.1
101	9.4	2.7	5.4	6.5	30				13
102	11	2	3.8	4.6	26	9			10
104	10.6	2	4.6	6	11	20	175	400	4.5
106	14.3	0.8	2.5	3.5	26	9			6
201	15.3	3	14	18	8	23	120	290	3
202	20.4	2.4	4.8	6.1	10		180	450	4.3
203	21	3.4	7.4	9.4	7	18	100		
1007	22	2.2	3.6	4.3	19	55	320	950	9
2057	24	2	4.5	5.4	9.5		155	360	4
RS280	27	1.6^a^	13^a^	29^a^	100	390			49
1601	9	240	240	240	0.16	0.8			
cvd3533	1.5	700	700	700	0.04		0.1		
cvd3540	1.5	20	30	41	1		30		
T1011	6	2.5	9.5	14	17	46		700	
T2015	5	6.1	15	21	7	16		250	
B73	200	0.83	1.3	1.4	63	220			30

^a^The two-layer model resulted in an unreasonable temperature dependence of *n* in sample RS280.

**Table 2:** Parameters in Eq. (3) describing the shape of PL bands in GaN.

PL band	Preliminary attribution	*ħω*_max_ (eV)	*S*_*e*_	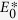 (eV)	ZPL at 18 K (eV)
RL1	?	1.78	10	2.33	—
RL2	?	1.75	23	2.49	—
YL1	C_N_O_N_? [6]	2.20	7.4	2.66	2.57
GL1	C_N_? [6]	2.40	8.5	2.92	—
GL2	V_N_ [19]	2.36	26.5	2.87	—
BL1	Zn_Ga_ [10]	2.9	3	3.17	3.10
BL2	C_N_H or C_N_O_N_H [18]	3.0	4.5	3.35	3.33

**Table 3:** Electron-capture coefficients (*C*_*nA*_, in 10^−12^ cm^3^/s) for the main PL bands in HVPE GaN samples.

Temperature	UVL (14)	BL1 (5)	GL1 (10)	YL1 (6)	RL1 (4)
100 K	3.2 ± 0.3	0.49 ± 0.06		0.11 ± 0.02	0.042 ± 0.010
180 K		0.39 ± 0.05		0.11 ± 0.02	0.037 ± 0.007
250 K			3.7 ± 0.5	0.12 ± 0.03	0.051 ± 0.012
300 K				0.11 ± 0.03	0.064 ± 0.010

The number of samples used in statistical analysis for a particular PL band is given in parentheses.

**Table 4:** The ratios of PL lifetimes at *T* = 100 K and calculated from these ratios coefficients *C*_*nA*_ in a set of 26 HVPE GaN samples.

PL band	UVL	BL1 (16)	YL1 (10)	RL1 (16)
*τ*_*nA*_/*τ*_*nUVL*_	1	4.70 ± 0.12	29.8 ± 1.2	74.4 ± 2.3
*C*_*nA*_ (10^−12^ cm^3^/s)	3.2 ± 0.3	0.68 ± 0.07	0.11 ± 0.01	0.043 ± 0.004

The number of samples used in statistical analysis for a particular PL band is given in parentheses.
